# Effects of Pgam1-mediated glycolysis pathway in Sertoli cells on Spermatogonial stem cells based on transcriptomics and energy metabolomics

**DOI:** 10.3389/fvets.2022.992877

**Published:** 2022-09-23

**Authors:** Xuejiao An, Qiao Li, Nana Chen, Taotao Li, Huihui Wang, Manchun Su, Huibin Shi, Youji Ma

**Affiliations:** ^1^College of Animal Science and Technology, Gansu Agricultural University, Lanzhou, China; ^2^Gansu Key Laboratory of Animal Generational Physiology and Reproductive Regulation, Lanzhou, China

**Keywords:** Sertoli cells, Pgam1, Spermatogonial stem cells, glycolytic metabolism, transcriptomics

## Abstract

Spermatogenesis is a complex process involving a variety of intercellular interactions and precise regulation of gene expression. Spermatogenesis is sustained by a foundational Spermatogonial stem cells (SSCs) and in mammalian testis. Sertoli cells (SCs) are the major component of SSC niche. Sertoli cells provide structural support and supply energy substrate for developing germ cells. Phosphoglycerate mutase 1 (Pgam1) is a key enzyme in the glycolytic metabolism and our previous work showed that Pgam1 is expressed in SCs. In the present study, hypothesized that Pgam1-depedent glycolysis in SCs plays a functional role in regulating SSCs fate decisions. A co-culture system of murine SCs and primary spermatogonia was constructed to investigate the effects of Pgam1 knockdown or overexpression on SSCs proliferation and differentiation. Transcriptome results indicated that overexpression and knockdown of Pgam1 in SCs resulted in up-regulation of 458 genes (117 down-regulated, 341 up-regulated) and down-regulation of 409 genes (110 down-regulated, 299 up-regulated), respectively. Further analysis of these DEGs revealed that GDNF, FGF2 and other genes that serve key roles in SSCs niche maintenance were regulated by Pgam1. The metabolome results showed that a total of 11 and 16 differential metabolites were identified in the Pgam1 gene overexpression and knockdown respectively. Further screening of these metabolites indicated that Sertoli cell derived glutamate, glutamine, threonine, leucine, alanine, lysine, serine, succinate, fumarate, phosphoenolpyruvate, ATP, ADP, and AMP have potential roles in regulating SSCs proliferation and differentiation. In summary, this study established a SCs-SSCs co-culture system and identified a list of genes and small metabolic molecules that affect the proliferation and differentiation of SSCs. This study provides additional insights into the regulatory mechanisms underlying interactions between SCs and SSCs during mammalian spermatogenesis.

## Introduction

Sertoli cells (SCs) are the only somatic cells in direct contact with spermatogenic cells within testis, SCs developing spermatogenic cells with structural, nutrient support. SCs also produce various cytokines and serve major component of Spermatogonial stem cells (SSCs) niche (1, 2). Because of their fundamental roles in spermatogenesis, any changes in SCs quantity and function likely affects the differentiation and self-renewal of SSCs. Sertoli cell-derived glial cell line-derived neurotrophic factor (GDNF), basic fibroblast growth factor (FGF2), and EGF (epidermal growth factor) regulate germ cell development and in culture, these factors are important for the establish and maintenance of SSC activity (3). For example, enhanced GDNF expression in SCs increased SSC proliferation (4). At present, the *in vitro* culture of sheep SSCs can only be carried out for preliminary isolation and short-term *in vitro* culture, and the purification method is not perfect, the purification effect is not ideal, and the *in vitro* culture system is not perfect. Therefore, in this study, mice were selected as model animals, and their SSCs were isolated, cultured and studied. In seminiferous tubules of mammalian testis, the self-metabolism of sperm cells cannot meet their energy needs, and it was proposed that SCs deliver glucose and other energy substances to germ cells (5). Lactic acid produced by SCs is not only an energy source for spermatocytes and sperm *in vitro*, but also inhibits the apoptosis of germ cells (6). After inhibiting the activity of mitochondria and supplementing of sperm flagellar oscillation and ATP production did not change; however, when sperm glycolysis was inhibited, sperm mitochondrial oxidation was then stimulated (7). Upon phosphorylation, sperm flagellar wiggle and ATP production levels were significantly reduced, suggesting that glycolytic metabolic process is one of the main sources of energy required for survival and differentiation of Spermatogenic cells (8). However, the effect and mechanism of action of glycolysis in SCs on SSC development has not been fully understood.

Energy metabolism (CCM), also known as central carbon metabolism, includes glycolysis pathway (EMP), tricarboxylic acid cycle (TCA) and pentose phosphate pathway (PPP). In addition, the enzymatic activities and protein expression levels of key enzymes in CCM are genetically distinguishable. In view of the important role of CCM in all living organisms, it is of great significance to study the expression of its metabolites. We previously isolated primary sheep SCs and studied the function of Pgam1 in SCs glycolysis, and found that Pgam1 can regulate SCs to produce lactate through glycolytic metabolism, which is an essential nutrient for spermatogenic cells and spermatogenesis (9). Because the functional interactions between SCs and SSCs is a necessary condition for spermatogenesis (10). Therefore, we speculate that Pgam1 may participate in the development and functional maintenance of SCs through glycolytic metabolism to produce lactic acid, an energy substrate required for the development of spermatogenic cells. In this study, we used mouse as research subjects and established a co-culture system of SCs-SSCs to explore the effect of Pgam1-mediated glycolysis metabolic pathway on SSCs mRNA expression profile and metabolic small molecules from the level of transcription and energy metabolism.

## Materials and methods

### Isolation of primary mouse SSCs

Isolation of primary mouse spermatogonial that containing enriched SSCs were conducted as previously described (11). Under sterile conditions, the newborn mice (5–7 days) were taken out, and the testes were sacrificed by cervical dislocation and placed in a petri dish with Dulbecco's Phosphate-Buffered Sallines (DPBS) (Gibco, New York, USA). The testis tissue was washed three times with DPBS, and the washed tissue was transferred to a 15 ml centrifuge tube, and gently pipetted with a 5 ml pipette until the tissue clumps became flocculent. Add an appropriate volume of collagenase I with a final concentration of 1 mg/mL and DNase I (Sigma Company, Shanghai, China) with a final concentration of 1 mg/mL, shake and digest at 37 °C for 5 min, and observe the frizzy state of the seminiferous tubules under a microscope. Put the digested tissue into a centrifuge at 20 g for 1 min, remove the supernatant, gently pipette with a pipette, and inoculate it on a petri dish. After culturing at 37 °C for 18–24 h, pour off the medium and add fresh culture medium. Then, the spermatogonia were gently blown off with a pipette, and the liquid was collected. After centrifugation at 600 g for 6 min, the supernatant was aspirated and the cells were resuspended with an appropriate amount of medium. All animals were managed according to the animal care and experimental procedure guidelines approved by the Animal Committee of Gansu Agricultural University (GSAU-AEW-2021-0017).

### Co-culture of SCs-SSCs

Using mouse SCs line (Beina Bio, Beijing, China) as feeder cells, the SCs cell line was recovered by conventional cell culture methods. After culturing for 48 h, the cells were digested, centrifuged and resuspended. The SCs medium was treated with mitomycin C (R&D Company, USA), and SCs were seeded at a density of 5 × 10^4^ cells/cm^2^ to prepare feeder cells. Primary stem cells were also seeded on feeder cells at a density of 5 × 10^4^ cells/cm^2^, and cultured by adding 20 ng/ml GDNF (R&D Company, USA) and 1 ng/ml bFGF (R&D Company, USA) at 37 °C and 5% CO_2_.

### Vector construction and cell transfection

The small interfering RNA (siRNA) sequence (Supplementary Table S1) targeting Pgam1 (SP group) the Pgam1 overexpression vector (pc-DNA-3.1(+)-Pgam1) (P group) were designed and completed by Genepharma (Shanghai, China) and Genewiz (Suzhou, China), respectively. The untransfected cells were used as the control group (NC group). The transfection began when the co-cultured confluence reached 50–70% 2,500 ng of the extracted Pgam1 overexpression plasmid DNA was diluted with 250 μL serum-free medium Opti-MEM (Gibco, New York, USA), and they were mixed gently; 5 μL Lipofectamine 2000 (Invitrogen, California, USA) was diluted with 245 μL serum-free medium Opti- MEM, and they were mixed gently, and incubated at room temperature for 5 min. Then the two liquids were mixed and incubated at room temperature for 20 min to form a DNA-Lipofectamine 2000 complex. After that, 500 μL of the complex was taken and added to 1.5 mL of Opti- MEM in a basic six-well plate. Then the two liquids were mixed gently placed it in a 37 °C incubator for 4 h, and then replaced with 15% FBS-containing DMEM/F12 medium for culture for 48 h. The transfection of siRNA dosage was 200 nM/well, and lipofectamine 2,000 dosage was 5 μL/well. The rest of the steps were the same as transfection plasmids. After transfection 48 h, as well as untransfected cells under the same culture conditions, were collected for transcriptome and energy metabolism sequencing in 3 replicates per group.

### RNA extraction and cDNA library construction

The SCs-SSCs cells of SCs 48 h after transfection were collected, washed twice with PBS, and 1 mL of Trizol was added to each well to extract the total RNA of the cells. The extracted total RNA samples were tested for purity and concentration by the spectrophotometer Nano Drop and bioanalyzer Agilent 2100, and SCs samples were used to detect overexpression and interference efficiency. High-quality RNA samples were used for cDNA library preparation and sequencing on the Illumina Hiseq 2500 platform.

### Transcriptome data processing and analysis

Quality control of the raw data obtained by sequencing was carried out, including removal of low-quality reads, to obtain high-quality clean data. Subsequently, clean reads were mapped to the mouse reference genome using TopHat2. The expression levels of mRNAs in each sample were normalized using the FPKM method (12). Differential expression analysis of the identified mRNAs between groups was performed using DEseq (https://cloud.metware.cn/#/tools/tool-list). |Fold change| > 2 and FDR < 0.05 were the conditions for the screening of differentially expressed genes (DEGs) among different groups. To determine their function, GO and KEGG enrichment analysis for DEGs was also conducted using the GO database (http://geneontology.org) and KEGG database (http://www.genome.jp/kegg/), respectively.

### RT-qPCR validation

To verify reliability of the transcriptomic profiling data, quantitative real-time PCR(RT-qPCR) was performed for 12 randomly selected DEGs. β-actin was used as a reference gene in quantitative analysis. An Evo M-MLV RT Kit with gDNA Clean for qPCR (Accurate Biotech, Hunan, China) was used to reverse-transcribe RNA into cDNA, RT-qPCR reactions were performed using SYBR Green Premix Pro Taq HS qPCR Kit (Accurate Biotech, Hunan, China) on the Roche LightCycler96. The relative expression was calculated using the 2^−ΔΔCt^ method. The qRT-PCR primers are listed in the Supplementary Table S2.

### Metabolomics sample handling and processing

The co-cultured cells were harvested 48 h after transfection. In total of 500 μL of 80% methanol/water (−20°C pre-cooled) extraction solution was added, and centrifuged at 2,500 rpm for 2 min. Cells were flash-frozen liquid nitrogen for 5 min and then thawing on ice for 5 min, repeating the above operation for 3 times. Then centrifuge at 12,000 rpm for 10 min at 4°C supernatant was transferred into a new centrifuge tube, and placed at −20°C for 30 min. After centrifugation, 200 μL supernatant was used for subsequent LC-MS analysis.

### Metabolomics data analysis

The sample extracts were separated by LC-ESI-MS/MS system using ACQUITY UPLC BEH Amide column (1.7 μm, 100 × 2.1 mm i.d), flow rate 0.40 mL/min, column temperature 40°C, injection Amount of 2 μL. Mobile phase composition A: ultrapure water (10 mM ammonium acetate, 0.3% ammonia water), B: 90% acetonitrile/water (V/V). Mobile phase gradient: 0–1.2 min A/B is 5:95 (V/V), 8 min A/B is 30:70 (V/V), 9.0–11 min A/B is 50:50 (V/V) V), 11.1–15 min A/B is 5:95 (V/V). The samples were subjected to mass spectrometry analysis using a Triple TOF 6600 mass spectrometer (AB SCIEX) after UHPLC separation.

### Enrichment analysis

The raw mass spectrometry data was converted into mzML format using ProteoWizard, use Analyst 1.6.3 software and MultiQuant 3.0.3 software to process the mass spectrometry data, use SVR to standardize and check the integrity of the data extracted by XCMS, and use the Peratoscaling method to analyze the data. Total peak areas are normalized. The processed data were input into SIMCA-P14.1 software for pattern recognition and multivariate statistical analysis, including principal component analysis (PCA) analysis, and supervised orthogonal partial least squares discriminant analysis (OPLS-DA). |Fold change| > 2 and VIP > 1 were used as the criteria for determining differential metabolites.

## Results

### RNA-seq data analysis

After filtering the low-quality reads, 1.44 million, 1.46 million, and 1.56 million high-quality sequences (clean reads) were obtained in the P, NC, and SP groups, respectively. The percentage of clean reads aligned to the mouse reference genome is between 92.51 and 96.17%, and about 85% of the clean reads are uniquely mapped (Supplementary Table S3).

### Gene expression analysis

**Figure 1:**
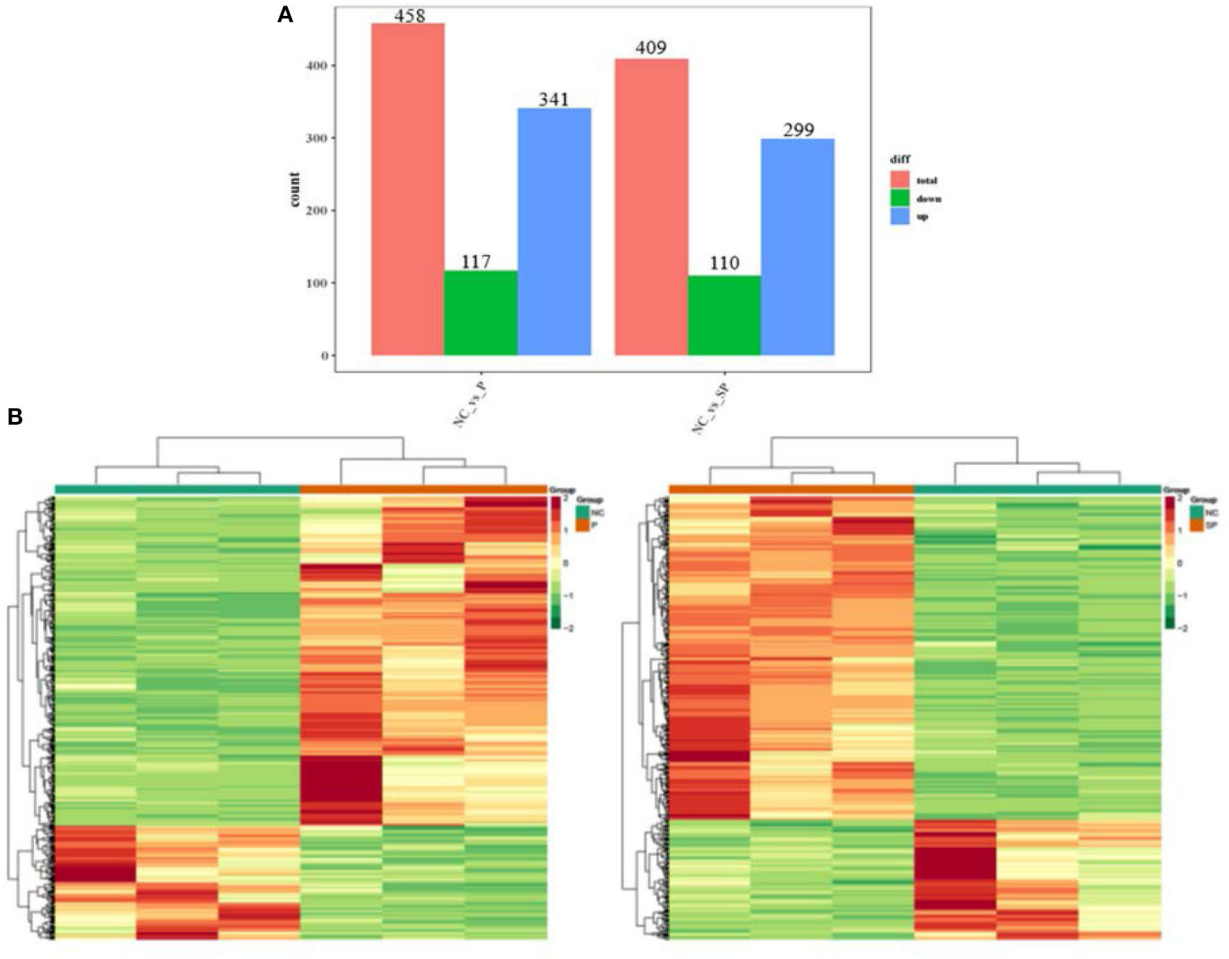
Analysis of DEGs. **(A)** Statistics of differentially expressed genes; **(B)** Clustering heat map of differentially expressed genes expression (red corresponds to upregulation, green corresponds to downregulation).

To investigate the differences in the transcriptional levels of mouse SCs-SSCs by perturbing or overexpressing Pgam1 gene, the transcripts of all differentially expressed genes were calculated using FPKM. In total 458 and 409 DEGs (|log_2_FC| > 1, FDR < 0.05) were identified in the NC vs. P and NC vs. SP groups, respectively. The bar graph reflects the number of up and down-regulated DEGs between the two comparison groups, where the number of down-regulated DEGs (117) was smaller than the up-regulated number of DEGs (341) after overexpression of Pgam1. After interfering with Pgam1, the number of up-regulated DEGs decreased from 341 to 299, and the number of down-regulated DEGs decreased from 117 to 110 (Figure 1A). In order to more intuitively show the differences and similarities between DEGs after overexpression and interference of Pgam1 gene, the expression of DEGs was analyzed with a cluster heat map, and it was found that there were significant differences in gene expression profiles between the two comparison groups (Figure 1B). The difference, that is, the genes with high expression in the P group were gradually down-regulated in the SP group, while the genes with low FPKM in the P group were gradually up-regulated in the SP group. This result indicates that overexpression of Pgam1 gene can inhibit the expression of one group of genes or induce the expression of another group of genes, and the changes of these genes may be to regulate the proliferation and differentiation of SSCs.

### Enrichment analysis

**Figure 2:**
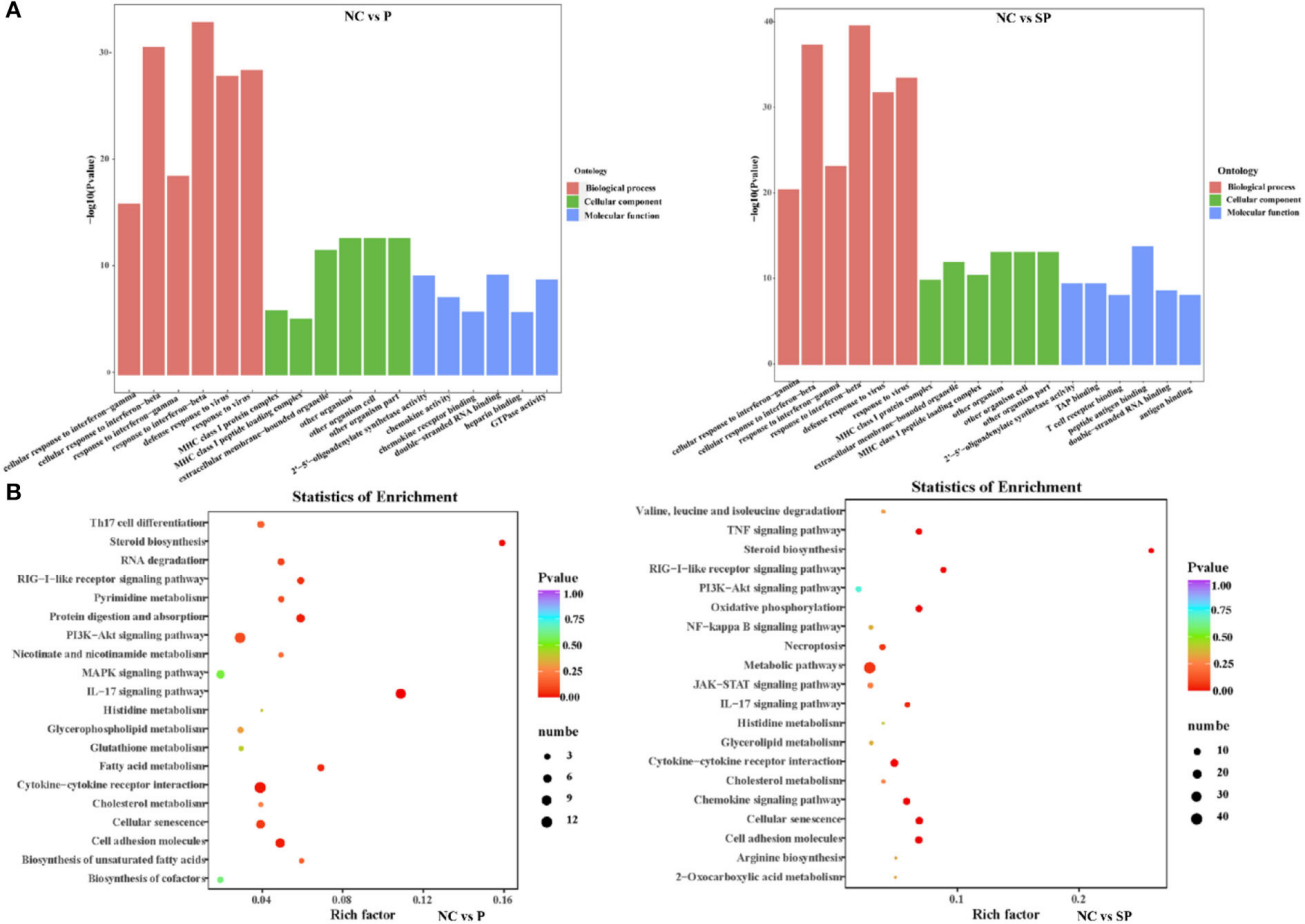
Analysis of GO and KEGG enrichment. **(A)** GO functional annotation of the differentially expressed genes in different groups. **(B)** KEGG pathway enrichment of the differentially expressed genes in different groups.

To further understand the biological functions of differentially expressed mRNAs, GO analysis was performed (Figure 2A). The results showed that most of the differential genes in the PGAM1 overexpression group, in biological process, were annotated to cellular response processes; in cellular component, were annotated to extracellular matrix components, MHC protein complexes, extracellular organelles, biological cells and other processes; in molecular function, were annotated to processes such as 2′-5′-oligoadenylate synthase activity, chemokine activity, chemokine receptor binding, double-stranded RNA binding, heparin binding, and GTPase activity. The differential genes following Pgam1 knockdown, in biological process, were also annotated to cellular response processes; in cellular component, were annotated to processes such as MHC protein complexes, extracellular organelles; in molecular function, were annotated to 2′-5′-oligoadenylate synthase activity, TAP binding, T cell receptor binding, peptide antigen binding, double-stranded RNA binding antigen binding processes.

To investigate whether DEGs in SCs-SSCs regulate the proliferation and differentiation of SSCs by participating in some specific pathways, KEGG pathway analysis was performed. Top20 of KEGG enrichment analysis showed that DEGs in NC vs. P group were mainly enriched in IL-17 signaling pathway, Cytokine-cytokine receptor interaction, Cell adhesion molecules, PI3K-Akt signaling pathway, MAPK signaling pathway, fatty acid, cholesterol glycerophospholipid, glutamate, glutathione metabolism. DEGs in NC vs. SP group were mainly enriched in Metabolic pathways, Cell adhesion molecules, Cellular senescence, Oxidative phosphorylation, Chemokine signaling pathway, Cytokine-cytokine receptor interaction and PI3K-Akt signaling pathway (Figure 2B).

### RT-qPCR verification of sequencing results

**Figure 3:**
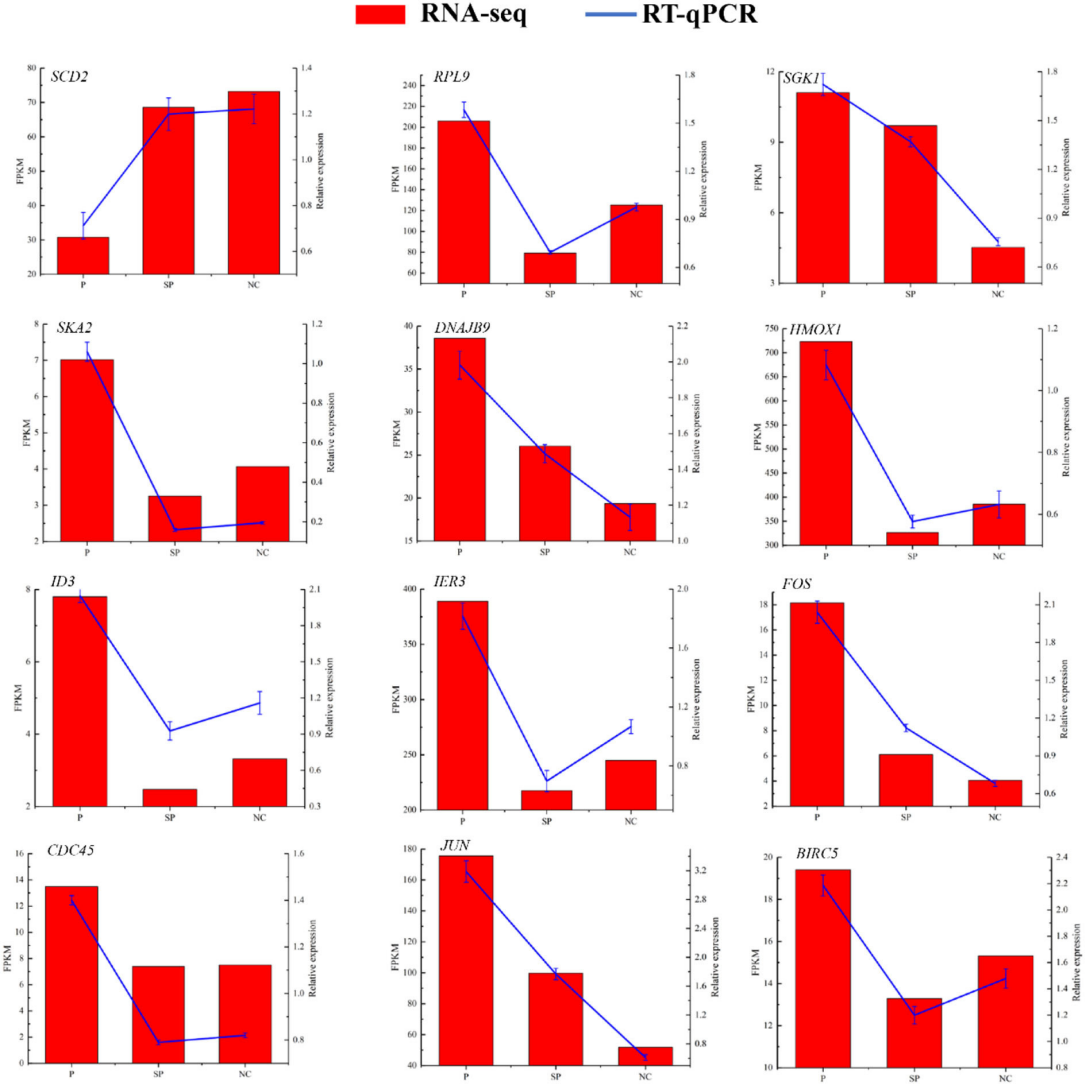
RT-qPCR to verify the expression pattern of DEGs in RNA-Seq. Histogram represent the relative expression level defense by qRT-PCR (right y-axis). Broken line indicates the change in transcript level according to the FPKM value of RNA-seq (left y-axis).

To verify the reliability of the sequencing data, 12 DEGs were randomly selected for RT-qPCR detection. As shown in Figure 3, their expression patterns detected with RT-qPCR were consistent with those acquired by RNA-Seq, indicating that the sequencing results were accurate and reliable.

### Overall sample PCA and cluster analysis

**Figure 4:**
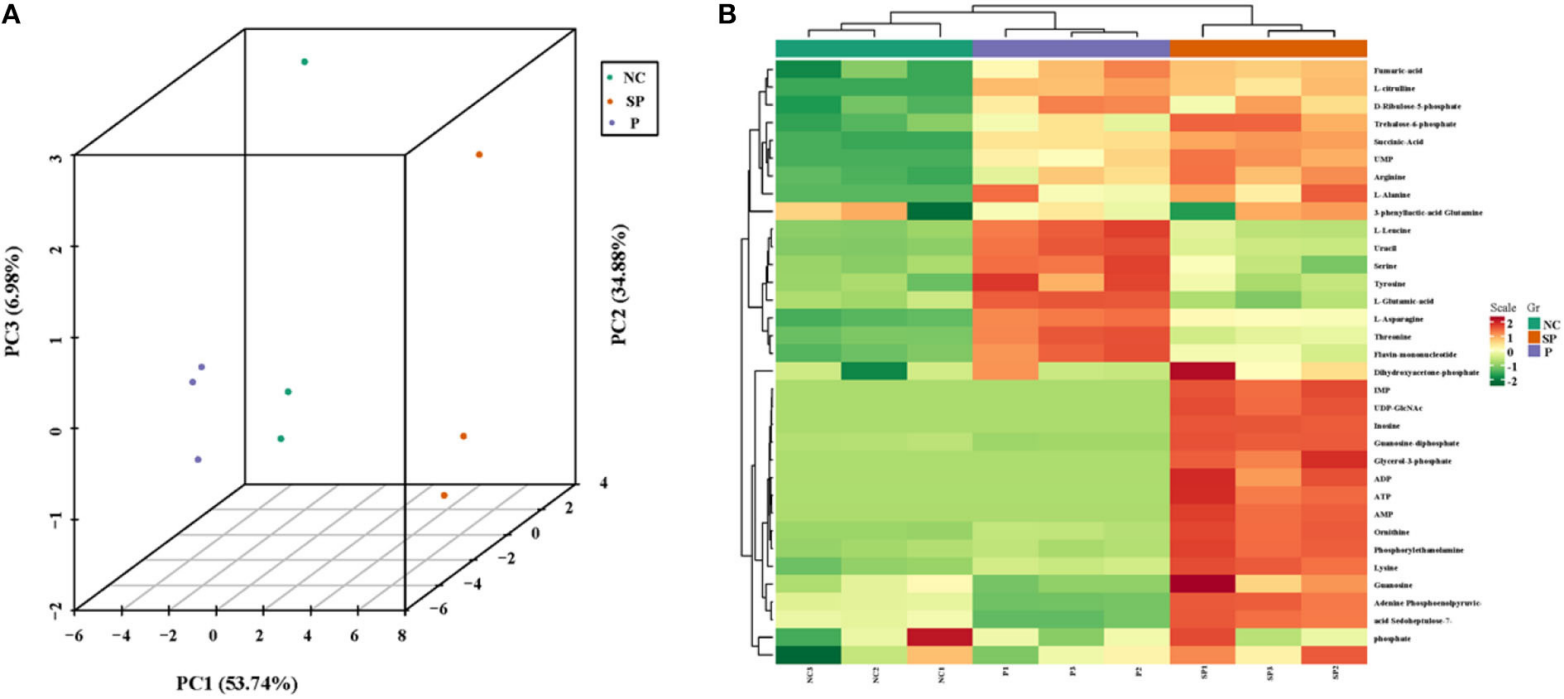
3D-PCA analysis diagram and heat map clustering. **(A)** 3D-PCA analysis diagram of three groups of samples; **(B)** Clustering heat map of three groups of differential metabolites.

As shown in Figure 4A, the samples are closely clustered in the 3D-PCA score map, indicating that the test has good repeatability and reliable data. Clustering heat map results showed that the cumulative pattern correlation heatmap among the selected three groups of samples was clearly divided into three branches (Figure 4B).

### OPLS-DA

**Figure 5:**
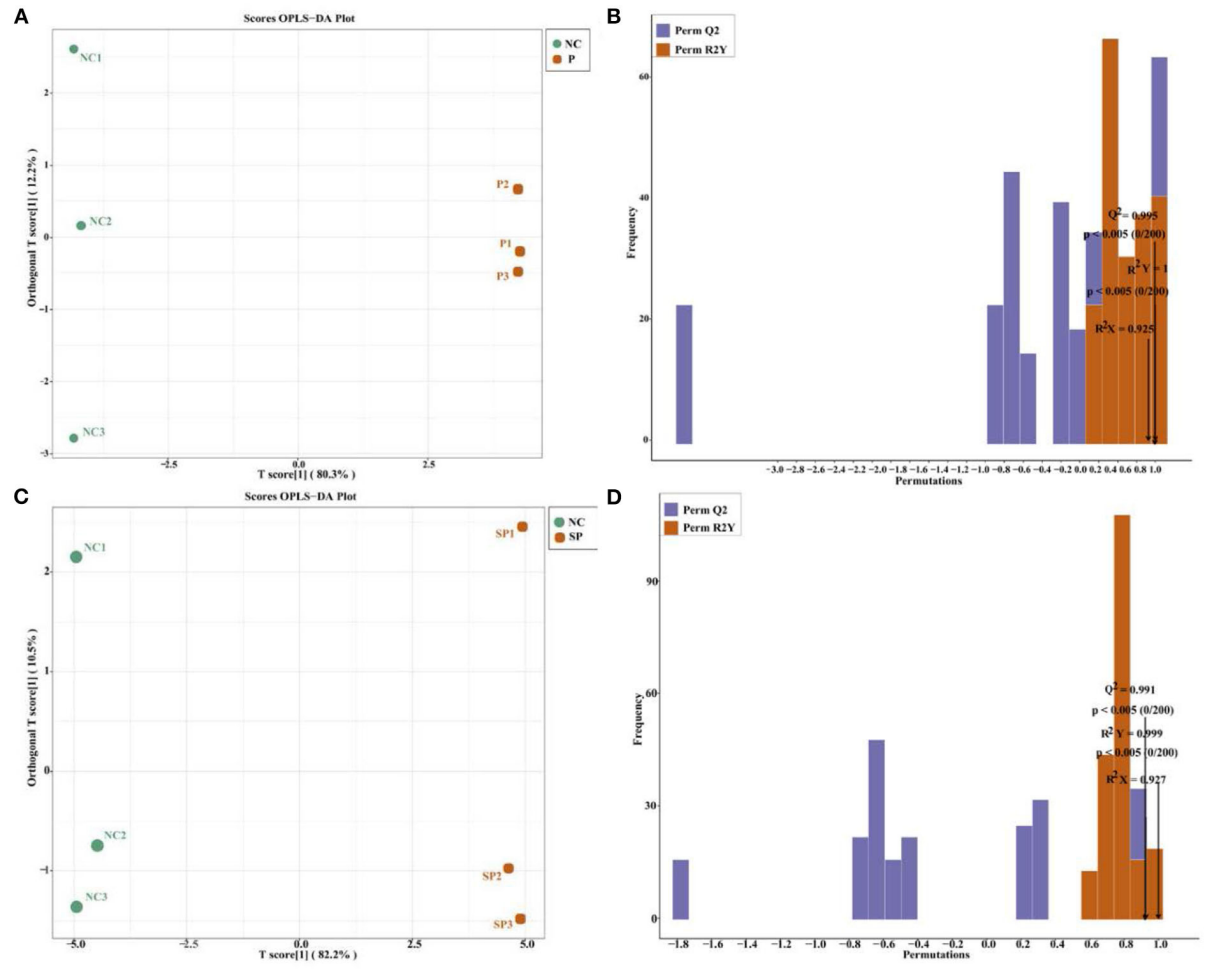
The score graph and verification graph of the OPLS-DA model. **(A)** NC vs. P OPLS-DA model score graph; **(B)** NC vs. P OPLS-DA model validation graph; **(C)** NC vs. SP OPLS-DA model score graph; **(D)** NC vs. SP OPLS-DA model validation graph.

It can be seen by OPLS-DA (Figures 5A,C) that the samples of each group were clearly separated, and it was found that the energy metabolites of mouse SCs-SSCs were all within the 95% confidence interval. Validation of the established OPLS-DA model found that the R2Y values of the NC vs. P and NC vs. SP models were 1 and 0.999, respectively, among the groups, the P values were all less than 0.05, and the Q2 values were all greater than 0.99 (Figures 5B,D), the above results show that the model established in this experiment is stable and reliable, and can be used for comparative analysis of differences between the two groups.

### Identification of differential metabolites

|Fold change| > 2 and VIP > 1 were used as the screening criteria for differential metabolites, and as a result, a total of 11 differential metabolites were identified in the PGAM1 gene overexpression group. Further analysis found that these 11 differential metabolites compounds belong to 6 substance categories: base acid derivatives, amino acids, phosphate compounds, nucleotides and their metabolites, organic acid derivatives and phosphate sugars. A total of 16 differential metabolites were identified in the PGAM1 gene interference group, and further analysis found that these 16 differential metabolites belonged to 5 substance categories, namely amino acids, lysophosphatidyl ethanolamine, phosphate compounds, nucleotides and their metabolites, organic acids and their derivatives (Figure 6).

**Figure 6:**
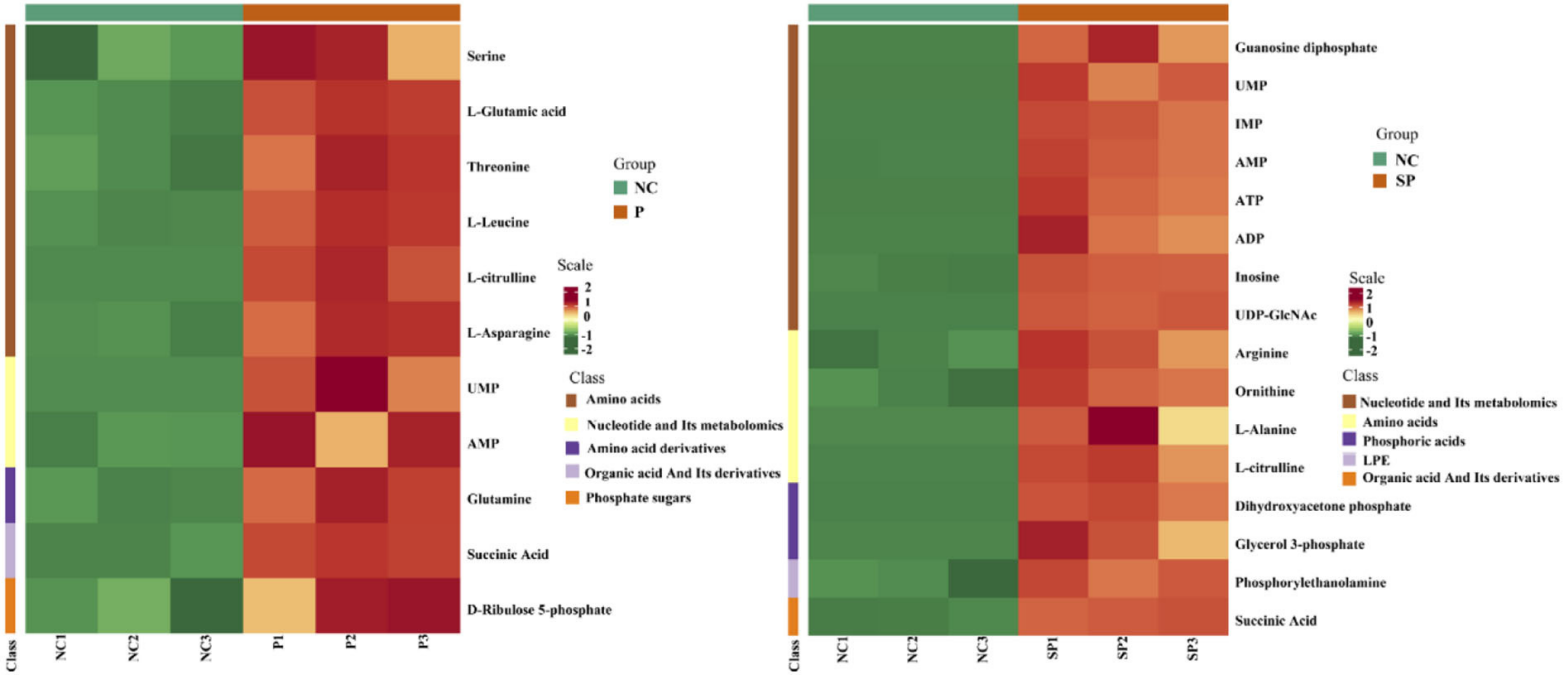
Cluster heat map of differential metabolites.

### Correlation analysis of differential metabolites

**Figure 7:**
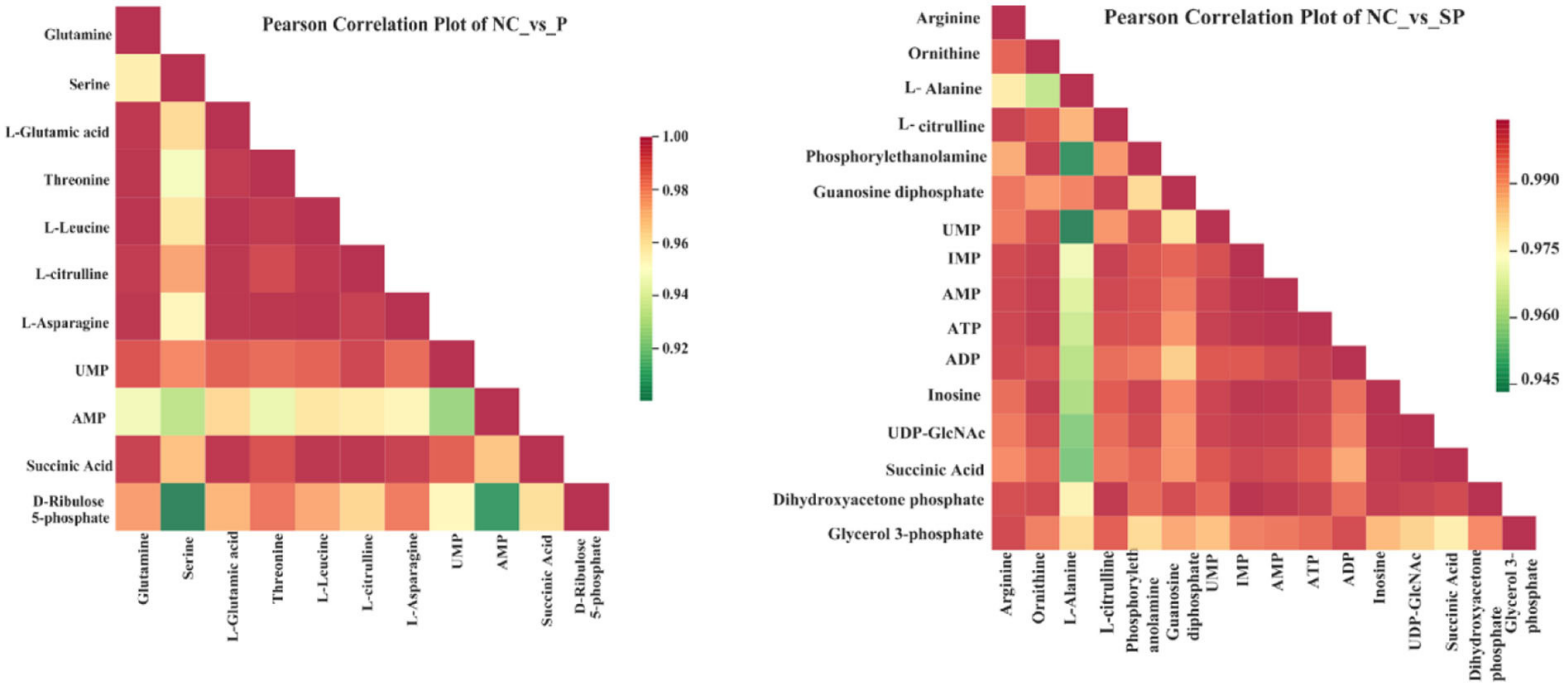
Correlation analysis diagram of differential metabolites.

Different metabolites have synergistic or mutually exclusive relationships, and correlation analysis can help measure the metabolic closeness between significantly different metabolites, which is beneficial to further understand the mutual regulation of Pgam1 through SCs to metabolites in SSCs. The results showed that in the Pgam1 gene overexpression group, glutamine was significantly positively correlated with other 10 metabolites; serine was significantly negatively correlated with AMP and D-ribulose 5-phosphate, and significantly correlated with other metabolites. Positive correlation; glutamate (glutamate) was significantly positively correlated with other metabolites; threonine, leucine, L-citrulline, L-asparagine were all associated with other metabolites Significant positive correlation; AMP and UMP were significantly negatively correlated, and significantly positively correlated with other metabolites. In the Pgam1 gene interference group, ornithine was negatively correlated with alanine, and positively correlated with other metabolites; alanine was positively correlated with phosphoethanolamine, UPMA, IMP, AMP, ATP, ADP, inosine, uridine diphosphate-N-acetylglucosamine and succinic acid were significantly negatively correlated, and significantly positively correlated with other differentially derived compounds; arginine, L-citrulline, guanosine diphosphate, dihydroxyacetone phosphate and 3- Glycerol phosphate was positively correlated with other metabolites (Figure 7).

### Pathway identification

**Figure 8:**
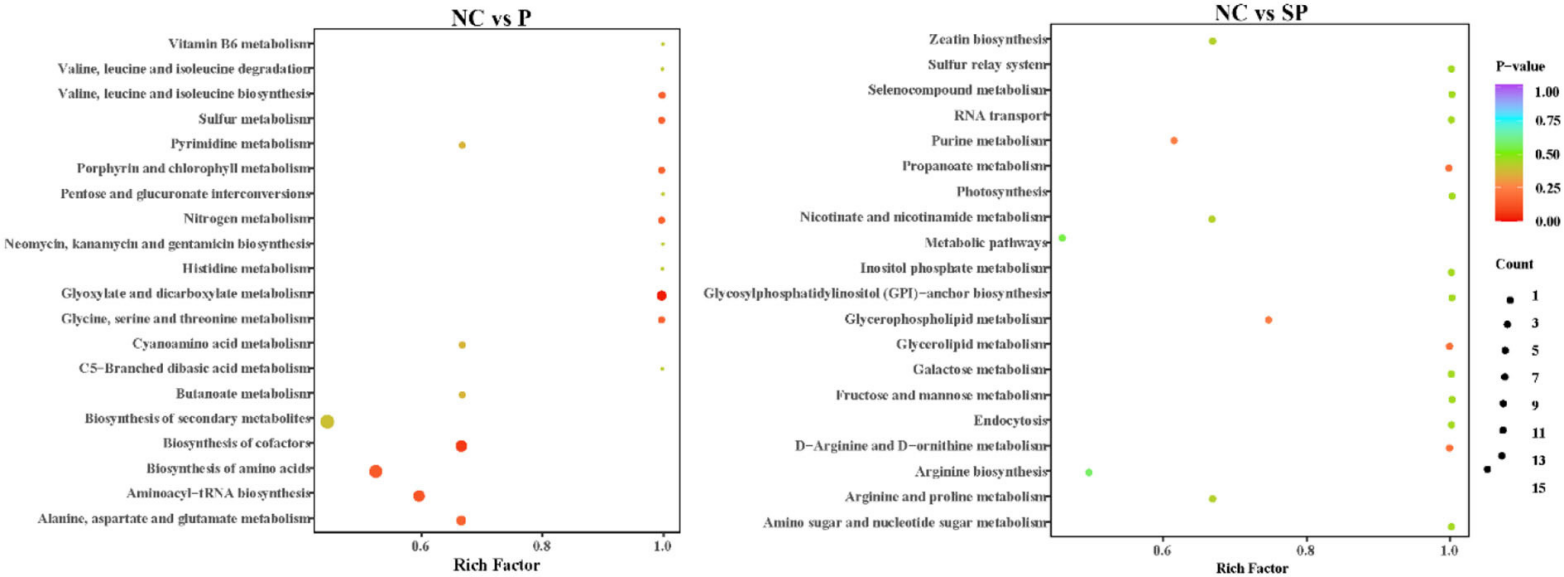
KEGG pathways significantly enriched in differential metabolites.

In this study, the KEEG annotation enrichment analysis results of differential metabolites are shown in Figure 8. In the Pgam1 gene overexpression group, the key differential metabolic pathways were glyoxylate and dicarboxylic acid metabolism, cofactor biosynthesis, aminoacyl-tRNA biosynthesis, amino acid biosynthesis, alanine, aspartate and glutamate metabolism, biosynthesis of secondary metabolites. In the Pgam1 gene interference group, the key differential metabolic pathways were glycerophospholipid metabolism, purine metabolism, metabolic pathway, arginine biosynthesis, oxidative phosphorylation, glyceride metabolism, D-arginine and D-ornithine metabolism and propionate metabolism.

### Conjoint analysis

**Figure 9:**
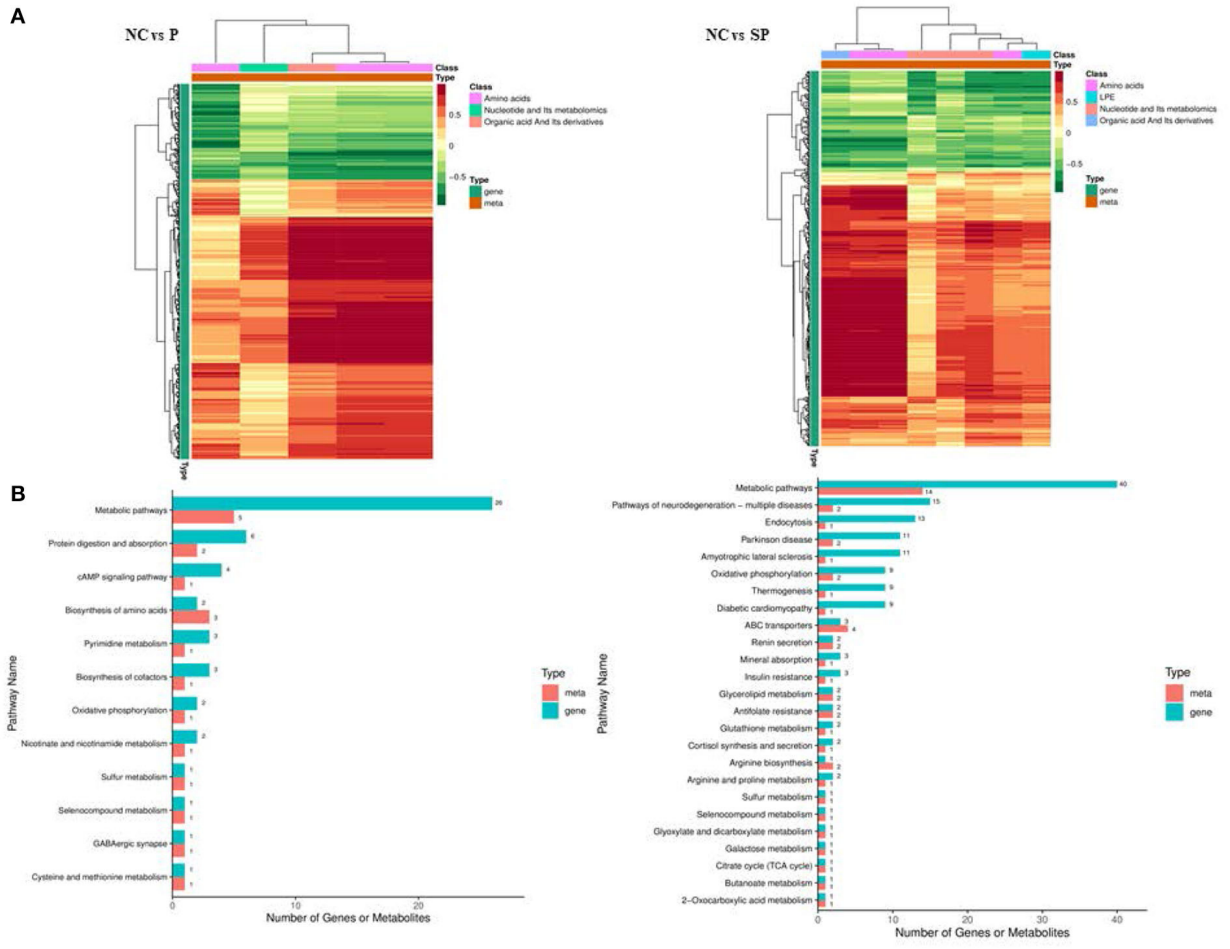
Combined analysis results of DEGs and differential metabolites. **(A)** Correlation cluster heatmap of differentially expressed genes and differential metabolites; **(B)** Bar chart of differentially expressed genes and differential metabolites KEGG enrichment.

Association analysis of differentially expressed genes and differential metabolites was performed based on transcriptome and energy metabolome results. As shown in Figure 9, amino acids, nucleotides and their metabolites, and organic acids and their derivatives in NC vs. P were significantly related to the DEGs screened in the transcriptome. Further research found that these DEGs and differential metabolites were mainly enriched in It integrates metabolic pathways, protein digestion and absorption, cAMP signaling pathway and amino acid biosynthesis. Amino acids, polyvinyl alcohol, nucleotides and their metabolites, and organic acids and their derivatives in NC vs. SP were significantly correlated with the DEGs screened in the transcriptome. Further research found that these DEGs and differential metabolites were mainly enriched in metabolic pathways, Pathways of Neurodegeneration - Pathways in various diseases, endocytosis, oxidative phosphorylation, thermogenesis, arginine biosynthesis, arginine and proline metabolism.

**Figure 10:**
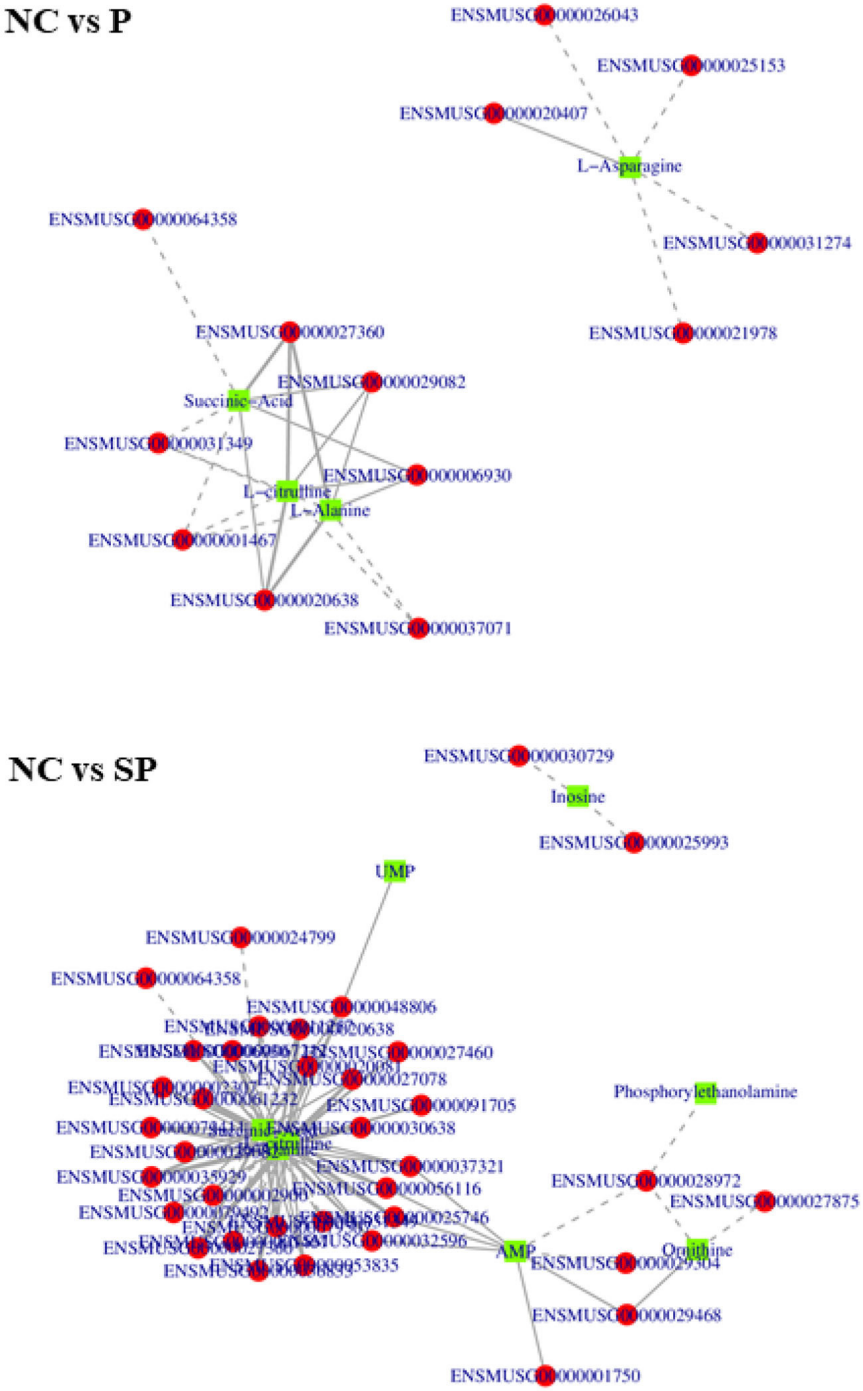
Correlation network diagrams of differential expressed genes and differential metabolites. The squares in the figure represent the differential metabolites, the circles represent the differential genes, the solid line represents the positive correlation, the dashed line represents the negative correlation.

In order to further study the regulation of these genes on differential metabolites, the correlation analysis between these differential metabolites and differentially expressed genes was performed. As shown in Figure 10, in NC vs. P, 8 genes were significantly correlated with alanine content., 6 genes were significantly associated with L-citrulline, 5 genes were significantly associated with L-asparagine content, and 7 genes were significantly associated with succinate content; In NC vs. SP, 1 gene was significantly related to uridine 5-monophosphate content, 2 genes were significantly related to inosine content, 8 genes were significantly related to AMP content, 8 genes were significantly related to ornithine count, 27 genes were significantly related to alanine, L-citrulline and succinic acidcontent.

In NC vs. SP, 1 gene was significantly associated with uridine 5-monophosphate content, 2 genes were significantly associated with inosine content, 8 genes were significantly associated with AMP content, and 8 genes were significantly associated with ornithine content There were 4 genes significantly related to the content of alanine, 27 genes significantly related to the content of L-citrulline, 27 genes significantly related to the content of L-citrulline, and 1 gene significantly related to the content of LPE; There were 27 genes significantly correlated with content.

## Discussion

GDNF in testis is secreted by SCs, and the self-renewal of SSCs is closely related to the amount of GDNF secreted. Studies have found that low levels of GDNF can lead to a decrease in the number of spermatogenic cells and abnormal spermatogenesis, while high levels of GDNF can cause excessive proliferation of undifferentiated spermatogonia (10). Fibroblast growth factor2 (FGF2) is a supporting factor for SSCs, and enables the expansion of SSCs *in vitro* (13). Some researchers directly introduced FGF2/GDNF signaling into mouse testis and found that both factors could induce the excessive proliferation of GFRα1+ undifferentiated spermatogonia (14). FGF2 can regulate ETS variant transcription factor 5 (ETV5) and GDNF mRNA expression by activating protein kinase (MAPK) and phosphatidylinositol 3-kinase (PI3K) (15). The study found that the addition of FGF2 to mouse SSCs cultured *in vitro* resulted in increased phosphorylation of MAP2K1 and expression of ETV5 and BCL6B transcription repressor BCL6B (BCL6B) genes; and after overexpression of MAP2K1 in mouse SSCs, the expression of ETV5 and BCL6B genes was also significantly increased. Thus, it is indicated that FGF2 promotes the self-renewal of SSCs by activating the MAP2K1 signaling pathway, thereby regulating the expression of ETV5 and BCL6B genes (16). In addition, some studies have found that FGF2 can also promote the *in vitro* proliferation of SSCs in the absence of GDNF, but with fewer proliferating cells. A previous study showed that the expression pattern of GDNF gene was basically the same as that of FGF2 in SSCs of cashmere goats at different prepubertal ages (17). It can be seen that FGF2 and GDNF act synergistically to jointly regulate the proliferation and apoptosis of SSCs (13). In this study, it was found that the expressions of FGF2 and GDNF were increased in the PGAM1 overexpression group, and decreased in the interference group. Combined with the results of previous studies, it is speculated that Pgam1 may cause the expression of FGF2 and GDNF in SCs cells to increase through the glycolysis pathway, thereby promoting the self-renewal of SSCs.

The PI3K-Akt signaling pathway can transmit extracellular stimuli through the cell membrane to the cytoplasm (18), and plays an important role in testicular development and spermatogenesis (19). Studies have found that PI3K/Akt is the main pathway for regulating glucose metabolism through insulin action. Activated PI3K can block the process of gluconeogenesis by inhibiting enolpyruvate carboxykinase, increase the utilization of glucose, and enhance PFK, which in turn increases the rate of glycolysis (20). At the same time, the activated Akt increases the intracellular ATP level, the expressions of GLUT, HK and PFK, and the activity of HK and PFK, and thus promoting the glycolytic metabolism (21). Inhibition of the PI3K/Akt signaling pathway results in the inhibition of glucose transport in the testis by insulin and IGF-I in rats (22). When PI3K-specific inhibitor was added to the medium of Germline Stem (GS)cells, it was found that the growth process of GS cells was significantly slowed down, and the number of cells did not increase after 6 days of culture. Therefore, it is suggested that the PI3K pathway plays a key role in the proliferation and differentiation of SSCs (23). Some studies have also found that the binding of GDNF to its receptor GFRα1 activates c-RET and binds to various molecules such as SHC, GRB2, PLC-γ, GRB7, and GRB10, and finally activates the PI3K-AKT and MAPK pathways (24). In this study, KEGG enrichment analysis found that many differentially expressed genes were enriched in the PI3K-Akt signaling pathway, which is a key pathway regulating the differentiation and self-renewal of SSCs. Therefore, it is speculated that the Pgam1-mediated glycolysis metabolic pathway of SCs may be activated by PI3K-Akt signaling pathway to regulate the differentiation and self-renewal of SSCs.

Glutathione (GSH) is a natural antioxidant that is abundant in animal body cells (25), and is also the main endogenous antioxidant that maintains redox homeostasis in sperm cells (26). GSH is synthesized from amino acids such as methionine, tryptophan, glycine, and glutamic acid. Studies have found that adding a dose of GSH to frozen dilutions or thaws of sperm can improve their quality, motility, and plasma membrane integrity (27). Researches show that methionine were used for the synthesis of cysteine through the sulfur transfer pathway, and then synthesize GSH in hepatocytes (28). About half of the intracellular GSH is synthesized from homocysteine (29). Supplementation of methionine in the diet of rats can significantly increase the content of GSH in the body (30). And in the process of culturing liver cells *in vitro*, the content of GSH in the cells is also significantly increased after methionine is added to the medium (31). The addition of melatonin to the medium of porcine SSCs cultured *in vitro* can increase the cell viability, and GSH count, and reduce the level of reactive oxygen species (ROS) in SSCs, indicating that t melatonin can scavenge intracellular ROS by increasing the content of GSH, thereby protecting cells from damage (32). Under normal physiological conditions, there is a certain concentration of ROS in SSCs to maintain the self-renewal and differentiation of cells. Studies in mice have found that an increase in intracellular ROS can inhibit the proliferation and differentiation of SSCs (33, 34). The production and scavenging of ROS in normal SSCs are in a state of dynamic equilibrium. The body senses and feedback regulate the state of ROS in SSCs through some transcription factors and enzymes, so that the intracellular ROS is in a relatively homeostatic state. Due to the aging of the animal body and changes in the surrounding environment, endogenous and exogenous ROS regulation disorders will be caused, which will destroy the homeostasis and cause oxidative stress damage to cells (35, 36). Some studies have found that ROS beyond the tolerance range of the body can cause a series of lesions in SSCs, and finally cause cell apoptosis and even necrosis (37). Our results found that the content of glutamate and glutamine was increased after overexpression of Pgam1, and was decreased after interference with Pgam1. Since glutamate and glutamine are precursors for the synthesis of GSH, the increase in their content makes GSH content is also elevated, and high GSH can scavenge the excess ROS generated by SSCs, thereby protecting cells from damage and increasing the cell viability of SSCs. In conclusion, Pgam1 gene may affect the activity of SSCs by regulating the changes of lactate content in the glycolytic metabolism of SCs to affect the content of GSH precursor substances in SSCs.

Glutamine as a nitrogen donor is a necessary condition for nucleotide biosynthesis, and it can also be used as a carbon source to support the energy metabolism of cancer cells. Once glutamine is deficient, it will cause cell death (38, 39). SCs can also use amino acids for energy, among which the oxidation of leucine and glutamine provides most of their energy, and other amino acids, such as valine and alanine, also play an important role in their energy metabolism. Glucose metabolism stimulates the conversion of valine to lipids, and glutamine inhibits the oxidation of leucine, valine, and alanine, but does not affect their conversion to lipids (40). Alanine is the main amino acid capable of neogenesis to glucose, which can be converted to pyruvate, which is then used by SCs as a substrate for the Krebs cycle and gluconeogenesis pathways. Alanine production and pyruvate consumption reflect the NADH: NAD+ ratio as well as the cytoplasmic redox state (41, 42). It was shown that after overexpression of Pgam1, the increase of amino acid content in SCs-SSCs not only regulates the content of ROS in SSCs, but also acts as an energy substrate to support energy metabolism and increase the survival rate of SSCs. The final step of the glycolytic pathway is pyruvate kinase catalyzing the production of phosphoenolpyruvate to pyruvate. Pyruvate kinase 2 is highly conserved in mammals and is related to cellular anabolism and is expressed in tumor cells (43), and higher expression in rapidly proliferating mouse tumor cells cultured *in vitro* (44). In this study, the content of phosphoenolpyruvate was higher in the overexpression group, indicating that overexpression of Pgam1 promoted the increase in the content of pyruvate during glycolysis and produced more lactate for the development of SSCs.

The energy metabolism is a basal function for maintaining specific biological functions of cells. Mitochondria are ubiquitous intracellular organelles that play important cellular functions including ATP production, and cell apoptosis. The ATP produced by mitochondrial oxidative phosphorylation is the energy directly utilized by life activities, and its rate is mainly regulated by the ADP/ATP ratio, the carboxylic acid cycle provides more reductases (45). During the metabolic process of cells, ATP, ADP, and AMP are transformed into each other, which changes the contents of the three high-energy compounds in cells, and finally leads to changes in the metabolic capacity of the body (46). In this study, we found that the contents of ADP and ATP were significantly decreased following Pgam1 knockdown, indicating that interfering with Pgam1 gene might reduce the transport processes of ATP and ADP between mitochondria and cytoplasm, inhibit the normal mitochondrial respiratory chain coupling and promote the release of mitochondrial apoptotic factors, thereby reducing the viability of SSCs.

## Conclusion

Based on the transcriptome data of overexpression and interference of Pgam1 gene in mouse SCs-SSCs, the genes and metabolites affecting the differentiation and self-renewal of SSCs were evaluated, of which many genes were found to be involved in PI3K-Akt signaling pathway, MAPK signaling pathway, metabolism pathway, fatty acid, cholesterol, glycerophospholipid, glutamate, glutathione metabolism and other signaling pathways. Among them, GDNF, FGF2 and other genes may be the key genes regulating the self-renewal of SSCs through Pgam1-mediated SCs glycolysis pathway. A total of 13 potential biomarkers were screened for energy metabolism. They were mainly enriched in arginine biosynthesis, alanine, aspartate and glutamate metabolism, amino acid biosynthesis, glyoxylate and dicarboxylic acid metabolism, metabolic pathways, oxidative phosphorylation and other pathways. These potential biomarkers and key metabolic pathways may be the target sites for Pgam1 to regulate the proliferation, apoptosis and differentiation of SSCs through SCs.

## Data availability statement

The mouse mRNA sequences in our manuscript have been deposited in the SRA of the NCBI, accession number is PRJNA874532.

## Ethics statement

The animal study was reviewed and approved by the Animal Committee of Gansu Agricultural University (GSAU-AEW-2021-0017).

## Author contributions

XA: conceptualization, methodology, software, formal analysis, and writing–original draft. QL, TL, and MS: methodology and software. NC: methodology and formal analysis. HW and HS: software. YM: conceptualization, writing-review & editing, supervision, project administration, and funding acquisition. All authors contributed to the article and approved the submitted version.

## Funding

This study was supported by the National Natural Science Foundation of China (31960662), Education Science and Technology Innovation project of Gansu Province (GSSYLXM-02), National Key R&D Program of China (2021YFD1100502), and Innovation Star project for outstanding graduate students of the Education Department of Gansu Province (2021CXZX-350). The funders had no role in study design, data collection and analysis, decision to publish, or preparation of the manuscript.

## Conflict of interest

The authors declare that the research was conducted in the absence of any commercial or financial relationships that could be construed as a potential conflict of interest.

## Publisher's note

All claims expressed in this article are solely those of the authors and do not necessarily represent those of their affiliated organizations, or those of the publisher, the editors and the reviewers. Any product that may be evaluated in this article, or claim that may be made by its manufacturer, is not guaranteed or endorsed by the publisher.
