# Supplementary material for: Effects of Pgam1-mediated glycolysis pathway in Sertoli cells on Spermatogonial stem cells based on transcriptomics and energy metabolomics
Source: Front Vet Sci. 2022 Sep 23;9:992877. doi: 10.3389/fvets.2022.992877 (PMC9540473; doi:10.3389/fvets.2022.992877)
Supplement: Supplementary file 1 [file Data_Sheet_1.ZIP › Supplementary Materials/Table S1.docx]

Table S1. Information of si-PGAM1 sequence

| Name | Sequence | |
| --- | --- | --- |
|  | sense（5'-3'） | antisense（5'-3'） |
| si-PGAM1 | GGUCUCAACAAAGCAGAAATT | UUUCUGCUUUGUUGAGACCTT |
| NC | UUCUCCGAACGUGUCACGUTT | ACGUGACACGUUCGGAGAATT |
