# Supplementary material for: Effects of Pgam1-mediated glycolysis pathway in Sertoli cells on Spermatogonial stem cells based on transcriptomics and energy metabolomics
Source: Front Vet Sci. 2022 Sep 23;9:992877. doi: 10.3389/fvets.2022.992877 (PMC9540473; doi:10.3389/fvets.2022.992877)
Supplement: Supplementary file 1 [file Data_Sheet_1.ZIP › Supplementary Materials/Table S2.docx]

Table S2. Information of primer sequence

| Gene | GenBank No. | Sequences (5’-3’) | Length/bp |
| --- | --- | --- | --- |
| β-actin | XM_024980020.1 | F: CTAGGCGGACTGTTACTGAGC  R: ATGTTTGCTCCAACCAACTGC | 239 |
| CDC45 | NM_001161623.2 | F: TATACGCTGGTTCCGGTTTC  R: TCTTCCTGTTTCGCTCCACT | 235 |
| RPL9 | NM_011292.2 | F: GACGTTCTCTCTTTGCCCCA  R: GTGATTGAAGTCCCTCCGCA | 152 |
| SGK1 | NM_001161845.2 | F: GATGGGCCTGAACGATTTTA  R: CCCTTTCCGATCACTTTCAA | 225 |
| ID3 | NM_008321.2 | F: GCCCGAGAGAAGGACTGAAC  R: CGACACCCCATTCTCGGAAA | 129 |
| FOS | NM_010234.3 | F: TACTACCATTCCCCAGCCGA  R: GCTGTCACCGTGGGGATAAA | 113 |
| SCD2 | NM_009128.2 | F: CGCCTTCCCCTACGACTACT  R: GGAACAGGAACTGCAAGACC | 201 |
| JUN | NM_010591.2 | F: GCACATCACCACTACACCGA  R: GGGAAGCGTGTTCTGGCTAT | 127 |
| IER3 | NM_133662.2 | F: CGGACTATGCGCTGGATCTT  R: GAAAGTCTCTGTCCGTCCCC | 222 |
| HMOX1 | NM_010442.2 | F: GAACCCAGTCTATGCCCCAC  R: GGCGTGCAAGGGATGATTTC | 117 |
| BIRC5 | NM_001012273.1 | F: TGAGCAAATTCTGCCCCCAG  R: CCATGTCCCCAAGCCTCTAC | 171 |
| SKA2 | M_025377.3 | F: AACTGCAGAGATTGGGCGAG  R: ATGCATCACGATCCTCTCGG | 85 |
| DNAJB9 | NM_013760.4 | F: TCTCGGATGCCAATAGTCGG  R: CTAGAACCATCCTGGCGTGT | 217 |
