# Supplementary material for: Effects of Pgam1-mediated glycolysis pathway in Sertoli cells on Spermatogonial stem cells based on transcriptomics and energy metabolomics
Source: Front Vet Sci. 2022 Sep 23;9:992877. doi: 10.3389/fvets.2022.992877 (PMC9540473; doi:10.3389/fvets.2022.992877)
Supplement: Supplementary file 1 [file Data_Sheet_1.ZIP › Supplementary Materials/Table S3.docx]

Table S3 The alignment statistics result with the reference gene for all samples

| Samples | Raw reads | Clean reads | GC Content (%) | Q30(%) | Mapped Reads |
| --- | --- | --- | --- | --- | --- |
| P | 59454772 | 57009542 | 49.99 | 92.89 | 54318981  (95.28%) |
| P | 46483056 | 44920202 | 51.69 | 94.04 | 42631503  (94.90%) |
| P | 44001724 | 42340216 | 51.8 | 94.3 | 40220758  (94.99%) |
| NC | 48075934 | 46354970 | 50.92 | 92.66 | 44656016  (96.33%) |
| NC | 49336298 | 44000722 | 50.71 | 93.51 | 40705682  (92.51%) |
| NC | 58816374 | 56073528 | 51.4 | 92.86 | 53937953  (96.19%) |
| SP | 62221180 | 60273104 | 51.91 | 92.75 | 57963097  (96.17%) |
| SP | 49514256 | 47488468 | 52.2 | 92.7 | 45627343  (96.08%) |
| SP | 47043334 | 45657746 | 50.83 | 92.65 | 43839568  (96.02%) |

Note: P, pcDNA3.1(+)-PGAM1 group; SP, si-PGAM1; NC, untransfected group.
